# The emerging roles and mechanism of m6a in breast cancer progression

**DOI:** 10.3389/fgene.2022.983564

**Published:** 2022-08-10

**Authors:** Mengying Zhou, Menglu Dong, Xue Yang, Jun Gong, Xinghua Liao, Qi Zhang, Zeming Liu

**Affiliations:** ^1^ Institute of Biology and Medicine, College of Life and Health Sciences, Wuhan University of Science and Technology, Wuhan, China; ^2^ Department of Thyroid and Breast Surgery, Tongji Hospital, Tongji Medical College, Huazhong University of Science and Technology, Wuhan, China; ^3^ Department of Biliary-Pancreatic Surgery, Tongji Hospital, Tongji Medical College, Huazhong University of Science and Technology, Wuhan, China; ^4^ Department of Plastic and Cosmetic Surgery, Tongji Hospital, Tongji Medical College, Huazhong University of Science and Technology, Wuhan, China

**Keywords:** breast cancer, M6A, methylation, gene expression, miRNA, metastasis

## Abstract

Breast cancer (BC) has continued to be the leading cause of cancer deaths in women, accompanied by highly molecular heterogeneity. N6-methyladenosine (m6A), a methylation that happens on adenosine N6, is the most abundant internal mRNA modification type in eukaryotic cells. Functionally, m6A methylation is a reversible modification process and is regulated by 3 enzymes with different functions, namely “writer”, “reader”, and “eraser”. Abnormal m6A modifications trigger the expression, activation, or inhibition of key signaling molecules in critical signaling pathways and the regulatory factors acting on them in BC. These m6A-related enzymes can not only be used as markers for accurate diagnosis, prediction of prognosis, and risk model construction, but also as effective targets for BC treatment. Here, we have emphasized the roles of different types of m6A-related enzymes reported in BC proliferation, invasion, and metastasis, as well as immune regulation. The comprehensive and in-depth exploration of the molecular mechanisms related to m6A will benefit in finding effective potential targets and effective stratified management of BC.

## 1 Introduction

Breast cancer (BC) has continued to be the leading cause of cancer deaths in women, and its incidence rates are still increasing globally (Sung et al., 2021). BC is a highly molecularly heterogeneous tumor type and is continually associated with headaches, such as recurrence, metastasis, and drug resistance (Yau et al., 2022) (Derose et al., 2011). These features cause certain bottlenecks in the diagnosis and treatment of BC. Therefore, exploring the molecular mechanism of the occurrence and development of BC, as well as the capability for the early diagnosis of BC, treatment monitoring, or the search for effective potential targets, is of great significance for the effective stratified management of BC and the development of new diagnosis and treatment methods (Ellis and Perou, 2013).

**FIGURE 1:**
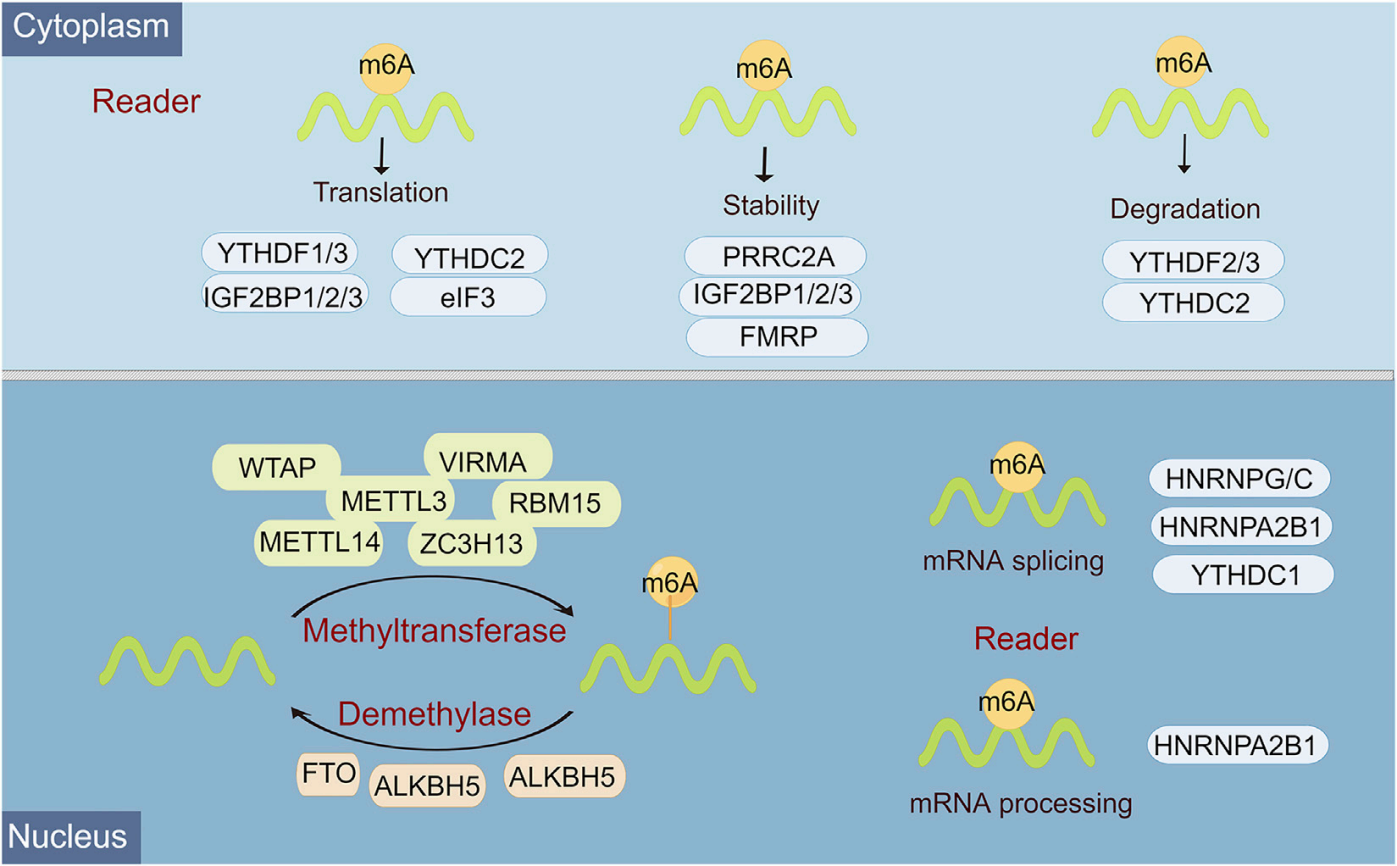
Overview of the classification and molecular mechanisms of m6A methylation. m6A RNA methylation is regulated by 3 different key enzymes, corresponding writers, erasers, and readers, which perform the functions of adding, deleting, or recognizing m6A, respectively. The consequences of m6A methylation lead to multiple processes in RNA metabolism and expression, including RNA splicing, miRNA processing, nuclear export, translation, stability, and RNA decay.

Epigenetic regulation, represented by N6-methyladenosine (m6A) modification, histone modification, DNA methylation, chromatin remodeling, and non-coding RNA (ncRNA) regulation, plays an overwhelming role in almost all biological behaviors, including cell differentiation and tissue development, and tumor progression (Wiener and Schwartz, 2021). M6A, a methylation that happens on adenosine N6, is the most abundant internal mRNA modification type in eukaryotic cells (Hu et al., 2022). There is a lot of evidence that m6A modification is an emerging important molecular modulation for tumors. In mammals, m6A modifications are known as a reversible dynamic process to influence different dimensions of RNA expression, including regulation of mRNA stability, splicing, translation efficiency, nuclear export, and degradation (Dierks et al., 2021). Functionally, this reversible m6A methylation modification is regulated by 3 enzymes with different functions, namely “writer”, “reader”, and “eraser” (Uddin et al., 2021). Among them, m6A can be installed by the methyltransferase complex, namely, writers, which include METTL3, METTL14, WTAP, RBM15/RBM15B, HAKAI, ZC3H13, and VIRMA/KIAA1429 (Huang et al., 2020). M6A eraser is able to remove methylation from m6A-modified RNA, mainly including FTO, ALKHB5, and ALKHB3 (Zhou et al., 2020). M6A reader is a selective RNA-binding protein that is responsible for catalyzing for recognizing m6A to activate downstream pathways, including YTH domain family 1-3 (YTHDF1, YTHDF1-2, YTHDF1), eIF3, IGF2BP1-3, hnRNPC, and hnRNPA2B1 (Dai et al., 2021) (Figure 1).

The enzymes involved in m6A modifications have been implicated in the regulation of gene expression and tumor evolution, including carcinogenesis, metastasis, and progression, especially in BC. For example, YTHDF1 overexpression is a not desirable signature for BC patients and is linked to lower immune infiltrate and poor clinical outcomes, while YTHDF1 inhibition promotes the proliferation, migration, and invasion in BC cell lines (Li et al., 2022). The results of sequencing data mining showed that m6A and its target genes have corresponding changes at the gene and protein levels in tumors, endowing the potential to indicate the prognosis of BC. In a bioinformatics analysis, high expression of IGF2BP1, a key m6A regulator, was often associated with shorter overall survival (OS) in BC patients. This suggests that IGF2BP1 is an independent prognostic factor in BC (Zhong et al., 2021). Besides, Chen et al. confirmed that METTL3 methylation is involved in KRT7-mediated m6A-induced BC lung metastasis (Chen et al., 2021). Furthermore, the expression profile of m6A regulators in BC is also prominently related to tumor malignancy, tumor immune score, anti-tumor immune response, and therapeutic effect (He et al., 2021). Gong et al. reported that METTL14 and ZC3H13 were positively correlated with the abundance of CD8^+^ T cells, neutrophils, macrophages, and dendritic cells (DCs) in BC.

Therefore, the systematic elucidation of the exact molecular mechanisms of m6A epigenetic regulation in BC progression is highly warranted. Here, we have reviewed and highlighted the roles of different types of m6A-related enzymes reported in BC proliferation, invasion, and metastasis, as well as immune regulation. Continued focus on the molecular mechanisms associated with m6A will benefit in finding effective potential targets and effective stratified management of BC.

## 2 The role of N6-methyladenosine modification in breast cancer progression

### 2.1 N6-methyladenosine writer in breast cancer progression

#### 2.1.1 METTL3

At present, METTL3 is the most studied methylation-modified protein that plays a broad regulatory role in BC progression. METTL3 mainly participates in the biogenesis, decay, and translation control of mRNA through m6A modification (Lin et al., 2016). Both METTL3 and METTL14 have methyltransferase activity, and the methyltransferase complex formed by the two performs catalytic function during the m6A process (Yankova et al., 2021).

In TNBC, METTL3 is an important collaborator in regulating metastasis, and low expression of METTL3 is implicated in the poor prognosis of triple-negative breast cancer (TNBC) (Shi et al., 2020). As Shi et al. confirmed, this metastasis-suppressing function of METTL3 was achieved by suppressing the expression of COL3A1 and its m6A function (Shi et al., 2020). METTL3 could accelerate the protein levels of SOX2, CD133, and CD44 to maintain or promote BC cell stemness, which was triggered by the m6A modification of SOX2 mRNA by METTL3, ultimately leading to the alteration in enhanced BC invasion and migration capabilities (Xie et al., 2021). In BC lung metastasis cell lines, m6A and methyltransferase METTL3 expression was enhanced, while the expression level of demethylase FTO was reduced (Chen et al., 2021). M6A was capable of regulating lung metastasis in BC cells by regulating m6A/KRT7/KRT7-AS. The study by Wang et al. revealed a similar conclusion that METTL3 was a tumor promoter and its knockdown could inhibit tumor progression by reducing methylation levels (Wang H. et al., 2020). This mechanism of action was achieved through the Bcl-2 pathway targeted by METTL3. In addition, hepatitis B X-interacting protein (HBXIP) was identified to promote METTL3 expression by repressing miRNA let-7g, while METTL3 was simultaneously able to induce HBXIP expression (Cai et al., 2018). This mechanism caused a positive correlation between the expression of METTL3 and HBXIP in BC tissues and a positive feedback regulation phenomenon.

In the process of BC cell behavior, ncRNAs represented by miRNAs, lncRNAs, and circRNAs have been identified as very important direct regulators of METTL3. Therefore, interactions mediated by METTL3 and ncRNAs regulate the expression levels of post-transcriptionally regulated genes that determine tumor fate. METTL3 was capable of influencing the malignant behavior of BC EMT. Specifically, inhibition of METTL3 diminished the m6A modification of MALAT1, subsequently downregulated the MALAT1 expression to suppress EMT in BC by sponging miR-26b to reduce the expression of HMGA2 (Zhao et al., 2021). Fan et al. demonstrated that LINC00675 was a tumor protective factor, and its low expression was associated with higher tumor grade, lymphovascular invasion, and shorter survival (Fan and Wang, 2021). Furthermore, *in vitro* studies indicated that LINC00675 inhibited BC progression by suppressing miR-513b-5p in a METTL3-related m6A-dependent manner. From clinical, cellular, and tumor-bearing mouse levels, Xu et al. demonstrated that zinc finger protein 217 (ZNF217) silencing or miR-135 elevation inhibited BC cell migration, invasion, and EMT initiation (Xu et al., 2022). This was mediated by a mechanism that, ZNF217 could upregulate NANOG by reducing m6A levels through METTL3, thereby forming a miR-135/ZNF217/METTL3/NANOG axis.

LINC00958 was an overexpressed lncRNA that promoted the malignant progression of BC tumors (Rong et al., 2021). And, LINC00958 bound to miR-378a-3p to regulate YY1 expression, on the other hand, METTL3-mediated m6A modification promoted LINC00958 expression upregulation. In TNBC cell lines, the overexpressed METTL3 was an accelerator to suppress the proliferation and invasion (Ruan et al., 2021). Further validation showed that circMETTL3 served as a sponge for miR-34c-3p and exerted tumor-promoting functions by upregulating the expression of METTL3. METTL3-derived circRNAs contributed to the proliferation and invasion of BC cells, through the competitive endogenous RNA (ceRNA) effect of miR-31–5p with upregulated CDK1 (Li et al., 2021). This function was also affected by the m6A modification mechanism of circMETTL3, which included METTL3.

#### 2.1.2 METTL14

METTL14 is an important RNA methyltransferase that serves an essential and significant role in the growth of tumors by regulating RNA expression. METTL14 has been demonstrated to be a core component of the m6A methyltransferase complex and is implicated in the dynamic and reversible process of m6A modification (Zhu X. et al., 2021).

LINC00942 (LNC942) might function as an oncogene that promotes BC cell proliferation, and colony formation and inhibits apoptosis (Sun et al., 2020). In BC cells, LINC00942 increased the METTL14-mediated m6A methylation and its associated mRNA stability, as well as the CXCR4 and CYP1B1 expression of CXCR4 and CYP1B1, revealing a novel LNC942-METTL14-CXCR4/CYP1B1 regulatory axis. Zhao et al. demonstrated that silencing of lncRNA UCA1 suppressed DNA methylation of RNA methyltransferase METTL14 (Zhao et al., 2022). This event promoted m6A modification of miR-375, leading to reduced SOX12 expression and eventually restrained BC proliferation and invasion. METTL14 is also an m6A methyltransferase that is significantly elevated in BC tissues. When METTL14 was overexpressed or its activity was inhibited, the invasive ability of tumor cells becomes enhanced or weakened, accordingly (Yi et al., 2020). The abnormal expression of METTL14 reconstructed the miRNA expression profile of BC cells, and mainly regulated the cell adhesion and invasion ability by regulating the expression of Hsa-miR-146A-5p.

#### 2.1.3 methyltransferase-like 5

Methyltransferase-like 5 (METTL5), can catalyze mA modification of 18S rRNA at adenosine 1832 (mA) in a critical position in the decoding center, possing the ability in regulating mRNA translation for impacting on cell growth (Sepich-Poore et al., 2022). In BC, METTL5 also exhibited a pattern of elevated expression and was required for the maintenance of BC cell lineage growth, reproduction, and S6K activation (Rong et al., 2020). The study by Rong et al. demonstrated that METTL5 was an 18S rRNA A1832-specific methyltransferase and was capable of regulating ribosome function via multiple models.

#### 2.1.4 KIAA1429

KIAA1429, also known as VIRMA, is recognized as the largest m6A methyltransferase and is employed as a scaffold for the catalytic core component of the bridging m6A methyltransferase complex (Lan et al., 2019). KIAA1429 plays an instrumental function in m6A modification and has previously been found to be dysregulated in a variety of cancer types. KIAA1429 is considered to be involved in BC carcinogenesis and progression. Zhang et al. showed that KIAA1429 was a significant promoter of tumor invasion and metastasis *in vitro* and *in vivo*, and affected the course of BC in a non-m6A-regulated manner (Zhang et al., 2022). KIAA1429 failed to interfere with m6A levels of SMC1A mRNA, implying that m6A modifications did not affect the interplay between KIAA1429 and SMC1A mRNA. KIAA1429 directly bound to the 3′-UTR of SMC1A mRNA, leading to the stability enhancement of SMC1A mRNA. KIAA1429 showed an interesting expression pattern with high expression in tumor entities but low expression in nontumorous tissues (Qian et al., 2019). In terms of prognosis, the high expression of KIAA1429 was often associated with a lower OS. Mechanistically, KIAA1429 plays a carcinogenic role in BC progression by regulating CDK1 in an m6A-independent manner. The detailed mechanisms of m6A writer in regulating BC progression could be seen in Table 1.

**TABLE 1:** The mechanisms of m6A writer in regulating BC progression.

Regulators	Expression pattern	Functions and mechanisms	Ref
METTL3	Low expression in TNBC	the low expression of METTL3-reduced m6A modification could promote TNBC metastasis by up-regulating COL3A1	(18)
METTL3	Upregulation in BC tissue, especially in T3-T4 or those accompanied with lymphatic metastasis	METTL3 promoted the stemness and malignant progression of BCa through mediating m6A modification on SOX2 mRNA	(19)
METTL3	Upregulation in BC tissue and cells	METTL3 knockdown could decrease the methylation level, reduce the proliferation, accelerate the apoptosis and inhibited the tumor growth by targeting Bcl-2	(20)
METTL3	Upregulation in BC tissue	HBXIP up-regulated METTL3 by suppressing let-7g, in which METTL3 increased HBXIP expression forming a positive feedback loop of HBXIP/let-7g/METTL3/HBXIP, leading to accelerated cell proliferation in BC	(21)
METTL3	Upregulation in BC tissue and cells	Silencing METTL3 down-regulated MALAT1 and HMGA2 by sponging miR-26b, and finally inhibited EMT, migration and invasion in BC	(22)
METTL3	−/−	METTL3 increased the m6A methylation of LINC00675, which enhanced the association between LINC00675 and miR-513b-5p	(23)
METTL3	−/−	MicroRNA-135 inhibited initiation of EMT in BC by targeting ZNF217 and promoting NANOG m6A modification	(24)
METTL3	Decreased in TNBC tissues and cell lines	circMETTL3 could act as a sponge for miR-34c-3p and inhibits cell proliferation, invasion, tumor growth and metastasis by up-regulating the expression of miR-34c-3p target gene METTL3	(26)
METTL3	−/−	circMETTL3 promotes BC progression through circMETTL3/miR-31–5p/CDK1 axis.	(27)
METTL14	Upregulation in BC cells and BC cohorts	LNC942 promoted METTL14-mediated m6A methylation in BC cell proliferation and progression	(29)
METTL14	Low expression in BC	LncRNA UCA1 promoted SOX12 expression by regulating m6A modification of miR-375 by METTL14 through DNA methylation	(30)
METTL14	Upregulation in BC tissue	METTL14 modulated m6A modification and hsa-miR-146a-5p expression, thereby promoting the migration and invasion of BC cells	(31)
METTL5	Elevated expression in BC tissue and cell lines	Ribosome 18S m6A methyltransferase METTL5 promotes translation initiation and BC cell growth, uncovering critical and conserved roles of METTL5 in the regulation of translation	(33)
KIAA1429	Overexpression in BC	KIAA1429/SMC1A/SNAIL axis in promoting EMT progress and metastasis in BC	(35)
KIAA1429	Highly expressed in BC tissues	KIAA1429 promoted BC progression and was correlated with pathogenesis by associating with CDK1 mRNA in an m6A-independent manner.	(36)

### 2.2 N6-methyladenosine Eraser in breast cancer progression

#### 2.2.1 Fat mass and obesity-associated

The fat mass and obesity-associated (FTO) gene is a well-known prominent factor in predicting obesity and is the first m6A eraser to be discovered in eukaryotic cells. FTO is responsible for controlling fatty acid transport, adipogenesis, fat metabolism, and obesity susceptibility. Single nucleotide polymorphisms (SNPs) of the FTO gene might be associated with various functions in different BC subtypes (Montazeri et al., 2022). It has been demonstrated that FTO expression is deregulated in a variety of tumors, including acute myeloid leukemia (AML), gastric cancer (GC), cervical squamous cell carcinoma (CSCC), ovarian cancer (OC), and BC (Deng et al., 2018).

As a key m6A demethylase, FTO is usually and aberrantly expressed up-regulated in BC tissues. High expression of FTO implies poor BC patient prognosis. Niu et al. determined that FTO remarkably contributed to BC cell proliferation and metastasis via the downregulation of tumor suppressor BNIP3, which involved FTO-mediated m6A demethylation in the 3′UTR of BNIP3 mRNA. Xu et al. demonstrated that in HER2-positive BC, the high FTO expression was linked to tumor progression, lymph node metastasis, TNM staging, and poor prognosis (Xu et al., 2020). *In vitro* experiments have similarly shown that FTO is a tumor-promoting factor that activates miR-181b-3p/ARL5B signaling leading to tumor migration.

#### 2.2.2 AlkB homolog 5

AlkB homolog 5 (ALKBH5) is another key m6A demethylase for gene transcription, translation, and metabolism, and is regarded as an effective biomarker for various diseases, especially cancers (Zhang et al., 2017). ALKBH3 preferentially acts on m6A in tRNA sites.

For instance, Wang et al. have previously reported that ALKBH5 could specifically regulate the function of AML leukemia stem cells without affecting normal hematopoietic stem cells (HSCs) (Wang J. et al., 2020). This highlighted the crucial role of ALKBH5 in maintaining cancer stem cell (CSC) renewal and cancer occurrence and development through the KDM4C-ALKBH5-AXL Signaling Axis. Hypoxia induces a series of stress changes to support the stable viability of cells, including changes in cytokine secretion profiles, and post-transcriptional and translational regulation. Reprogramming of the mA epitranscriptome is vital for the formation of the transcriptome and proteome in the setting of hypoxia (Wang et al., 2021). Similarly, Zhang et al. showed that hypoxia-induced an enhancement of NANOG mRNA and protein expression and breast CSC phenotype in a HIF- and ALKBH5-dependent manner, and that ALKBH5 deficiency was a debilitating factor for the hypoxia-induced BC CSC enrichment (Zhang et al., 2016).

### 2.3 N6-methyladenosine Reader in breast cancer progression

#### 2.3.1 YTHDF1

YTHDF1-3 are the three major m6A binding proteins and the most widely studied and versatile m6A readers (Chen et al., 2019). YTHDF1-3, containing special YTH domains, possess the capabilities of targeting and recognizing m6A-modified RNAs and mediating their degradation. YTHDF1 is a typical and highly expressed m6A reader protein in BC tissues and cell lines, and its high expression status is thought to be indicative of tumor size, metastasis, poor prognosis, and chemotherapy resistance (Anita et al., 2020). YTHDF1 is involved in almost the whole process of tumor biological behavior, and plays an important role in regulating transcription, translation, protein synthesis, angiogenesis, and EMT. Intriguingly, YTHDF1 as a target of tumor immune regulation has also attracted much attention.

Chen et al. showed that YTHDF1 promoted YTHDF1/FOXM1 to enhance FOXM1 expression, which in turn intensified the proliferation, invasion, and EMT phenotype of BC cells (Chen et al., 2022). Another 2022 similar study showed that a series of cascade reactions triggered by YTHDF1 were important molecular events in driving BC. Specifically, HIF1α expression could be induced and miR-16-5p levels were suppressed in a hypoxic microenvironment, resulting in upregulated YTHDF1 expression (Yao et al., 2022). Also, inhibition of YTHDF1 was able to promote the down-regulation of the glycolytic gene PKM2 to reduce BC glycolytic activity and lead to tumorigenicity and metastasis inhibition. These studies suggest that YTHDF1 can be used in a variety of regulatory pathways to modulate BC progression, and that depletion or targeted inhibition of YTHDF1 is a potentially efficient BC therapeutic strategy.

#### 2.3.2 YTHDF2

YTHDF2 is an N-methyladenosine-binding protein and can modulate mRNA stability, thus impacting central nervous system responses, embryonic development, and tumor evolution. Tumor biology studies have shown that YTHDF2 can modulate m6A modification to regulate downstream signaling molecules to regulate tumor cell proliferation, invasion, and migration (Shen et al., 2021). For instance, SUMOylation of YTHDF2 promotes mRNA degradation and cancer progression by increasing its binding affinity to m6A-modified mRNA (Hou et al., 2021). Einstein et al. uncovered a mechanism associated with RNA-binding proteins (RBPs), that the suppression of YTHDF2 initiated proteotoxic cell death pattern in MYC-driven TNBC (Einstein et al., 2021). This work not only demonstrated that YTHDF2, aberrantly expressed RBP and its mode of interaction with RNA were essential for BC cell growth, but that targeting YTHDF2 and specific RBP possessed outstanding BC therapeutic potential. The important mechanism of post-translational protein modification of YTHDF2, fully confirmed that YTHDF2 has a delicate manipulation between the regulation of protein post-translational modification and RNA chemical modification.

#### 2.3.3 YTHDF3

YTHDF3, in combination with YTHDF1 and YTHDF2, has a crucial effect in enhancing the synthesis of m6A-modified mRNAs in the cytoplasm (Shi et al., 2017). In TNBC subtypes, YTHDF3 expression was associated with poorer disease-free survival (DFS) and overall survival (OS) in patients (Lin et al., 2022). YTHDF3 could intensify the ZEB1 mRNA stability in an m6A-dependent manner, consequently leading to BC cell growth and EMT (Lin et al., 2022).

Intriguingly, the high expression level of YTHDF3 was also closely related to the prognosis of patients with breast cancer brain metastases (BCBMs). By enhancing the translation of m6A-enriched transcripts of ST6GALNAC5, GJA1, and EGFR, YTHDF3 promoted the communication between BC cells, endothelial cells, astrocytes, and tumor metastasis phenotypes represented by angiogenesis (Chang et al., 2020). Therefore, YTHDF3 could affect cascade steps in the BCBM, and then domesticate the evolution of BC cell changes in TME toward inducing brain metastatic polarity.

Totally, different binding proteins selectively recognize m6A-modified RNAs for achieving gene expression regulation. YTHDF1 is conducive to the enhanced mRNA translation, YTHDF2 is responsible for mRNA degradation, and YTHDF3 precipitates in the translation and degradation via the reciprocity actions with YTHDF1 and YTHDF2. This means that YTHDF3 can both collaborate with YTHDF1 to catalyze the translation of methylated RNAs, or directly engage with YTHDF2 to accelerate the decay of mRNAs. Thus, YTHDF1, YTHDF2, and YTHDF3 exert irreplaceable functions to foster BC progression and potentially even become robust therapeutic targets for prognostic stratification and effective treatment of BC.

#### 2.3.4 IGF2BP1

As a post-transcriptional fine regulator, IGF2BP1 plays a role in remodeling tumor growth, chemotherapy resistance, and macroscopically, OS and recurrence of tumor patients. GF2BP1 potentiates tumor malignant progression in a variety of solid tumors and exhibits a poor prognostic indicative value (Glaß et al., 2021). The principal action of IGF2BP1 in oncogenic cells is to stabilize mRNA encoding oncogenic factors. In pan-cancer studies, the high expression and tumor-promoting characteristics of IGF2BP1 in specific tumors make it a promising therapeutic target, but it is also inhibitory in some tumors (Huang et al., 2018).

Zhu et al. identified a hypoxia-induced lncRNA KB-1980E6.3, that exhibited abnormal BC tissue upregulation and was associated with a poor prognosis (Zhu P. et al., 2021). LncRNA KB-1980E6.3 increased the stability of c-Myc mRNAs by binding to m6A reader IGF2BP1 and consequently maintained the stemness of BCSCs. Interrupting this mechanism was of the potential to provide a therapeutic strategy for hypoxic tumors. MIR210HG acts as an oncogenic lncRNA highly expressed in BC tissue, and could promote BC metastasis, by inhibiting its encoded miR-210 (Shi et al., 2022). Moreover, MYCN directly activated IGF2BP1, and both IGF2BP1 and ELAVL1 strengthened the MIR210HG stability, resulting in a MYCN/IGF2BP1/MIR210HG regulatory axis.

## 3 N6-methyladenosine modification in immune regulation

Immune cells, secreted factors, and the tumor immune microenvironment in which they are intertwined are indispensable key links in the anti-tumor response (Liu et al., 2021). Considerable evidence suggests that m6A is involved in processes that regulate innate and adaptive immune cells, which in turn have been assigned roles in anti-inflammatory, anti-infective, and anti-tumor immunity (Ma et al., 2021). There is also much literature based on the existing reported m6A regulators, mining the correlation and prognostic scoring model of m6A regulators and immune infiltrating cells, for providing a novel evaluation tool for the diagnosis, prognosis, and immune status of BC (Yuan et al., 2022).

The immunomodulatory role of METTL3 in BC has been frequently reported. Yin et al. found that knockdown of METTL3 in bone marrow cells triggered malignant tumor proliferation and metastasis and exhibited an elevated abundance of M1/M2-like tumor-associated macrophages (TAMs) and Treg infiltration (Yin et al., 2021). Mechanistic studies suggested that deletion of METTL3 disrupted YTHDF1-mediated SPRED2 translation, thereby enhancing NF-kB and STAT3 activation via the ERK pathway, leading to tumor progression. Meanwhile, as the therapeutic benefit of programmed cell death receptor 1 (PD-1) inhibitor was weakened in Mettl3−/− mice, METTL3 could be a potential target for tumor immunotherapy. In addition, it has been reported that the expression of programmed cell death 1 ligand (PD-L1) was positively linked to the expression of METTL3 and IGF2BP3 in BC tissues (Wan et al., 2022). Since METTL3-mediated m6A modification could enhance PD-L1 mRNA stability through the METTL3-IGF2BP3 axis, tumor immune cell infiltrations and CD8^+^ T cell functions were enhanced forcefully when METTL3 or IGF2BP3 is inhibited. Ou et al. identified a specific C5aR1+ neutrophil subpopulation that potentiated BC cell glycolysis through ERK1/2-WTAP-ENO1 signaling, indicating that C5aR1+ neutrophils and the associated WTAP-ENO1 axis contribute to potential BC therapeutic target (Yin et al., 2021).

Another study reported that the co-expression network of YTHDF1 is critical in shaping immune responses, including antigen processing and presentation (Hu et al., 2021). YTHDF1 may act as a hopeful pan-cancer immune biomarker, as well as a novel promising marker for tumor immunotherapy. These results provide strong evidence that m6A modification is involved in the complex immune regulation of BC. M6A modifications can reshape antitumor immune responses by affecting immune cell state and function, and post-transcriptional regulation of specific cytokines and proteins.

## 4 Discussion

Based on these current developments, it is evident that m6A plays a dual role in shaping tumor progression. Specifically, m6A regulates the expression of its target genes to influence tumor progression, and whether the target genes act as tumor promoters or tumor suppressors determines the tumor-promoting or tumor-suppressing function of m6A. The presence of m6A modifications contributes to the promotion/suppression of various cellular functions, such as precursor mRNA splicing, nuclear translocation, stability, translation, and microRNA biogenesis, as the modification represented by tumor cells and immune cells, thus remodeling the BC progression.

In terms of BC diagnosis, m6A enzymes have also demonstrated good predictive efficacy. This is due to the fact that m6A enzymes show a characteristic pattern of differential expression in different BC subtypes and BC staging classifications. There have been more than 20 risk models based on these screened m6A regulators in BC research. For example, the overexpression of YTHDF1, YTHDF3, and KIAA1429 predicted a poor prognosis in terms of overall survival (OS), and the upregulation of YTHDF3 was an independent prognostic factor for OS in BR patients (Liu et al., 2019). There is even a strong performance of m6A enzymes in the treatment efficacy and recurrent metastasis of BC.

Aberrant expression of m6A regulators, are potential indicators for BC prediction, individually or synergistically. Other reported factor-based (such as ferroptosis, autophagy, lncRNA, m6A regulator-mediated immune Genes) models are also of huge value that have been confirmed in multiple studies. He et al. constructed an m6A regulator pattern, which could be effective for predicting malignancy, outcomes, and antitumor immune response (He et al., 2021). Moreover, the established models based on both m6A and other factors are attracting more and more attention. For instance, we previously constructed a risk signature based on 6 screened m6A-related lncRNAs, including Z68871.1, AL122010.1, OTUD6B-AS1, AC090948.3, AL138724.1, EGOT (Lv et al., 2021). This model could identify the prognosis and immune state in BC. Zhang et al. also adopt 21 m6A-related lncRNAs to establish a predictive model for predicting prognostic situations and BC subtypes with different immunogenicity (Zhang et al., 2021). Most of these models tend to build more accurate and extensive prediction models based on the inclusion of M6A-related indicators, thus providing rich information on tumor malignancy, tumor metastasis and recurrence, immune microenvironment, efficacy monitoring, and drug resistance evaluation.

Moreover, m6A-related models may have superior diagnostic value by associating with other models, including death (necroptosis, autophagy, and ferroptosis) genes, immune genes, glycosylation genes, and so on. However, since most of these are studies based on database excavation, they are retrospective studies. There are still relatively few prospective studies that can actually be performed in a large sample of realistic cohorts, and need to be further corroborated in actual clinical practice to prove their credibility. Overall, as a single regulator included in the model can offer many disease information, more gene combination models potentially provide a more comprehensive for breast cancer diagnosis and therapeutic outcomes. We must first admit that the reported single or the risk models based on these screened m6A regulators, is not the replacement for traditional and classical methods such as serology, pathology, and imaging. To be more precise, the traditional multi-method routine is the gold standard for diagnosis and prediction, while the risk models based on these screened m6A regulators are potential multifactorial predictors, which can be used as a useful supplement to clinical routine evaluation methods.

In terms of m6A-targeted BC tumor therapy, the following points still deserve in-depth consideration. First, m6A can regulate tumor progression through multiple mechanisms, which has been confirmed in BC. How m6A modification dysregulation is regulated by affecting tumor stem cells, immune environment, tumor cell fate, and other multiple ways is an important theoretical basis for strengthening m6A as tumor therapy. However, the role of m6A is dual in tumors, and a comprehensive assessment of how to regulate m6A-related enzymes by inhibition or activation requires specific BC subtypes, tumor microenvironment, and other underlying diseases. Moreover, ncRNA is an important player involved in tumor regulation, and how m6A RNA modification affects ncRNA function deserves further exploration.

Secondly, the primary prerequisite for targeting m6A-modified enzymes is the resolution of the protein crystal complexes of these enzymes in order to mine and design docked high-affinity small molecules and antibodies by conformational relationships (Oerum et al., 2021). These inhibitors and antibodies, in turn, will be considered to provide a good pre-requisite for clinical targeting only after effective validation in cellular and animal experiments (You et al., 2022). Finally, numerous studies have explained the critical role of m6A recognition proteins in BC, but a considerable number of m6A regulators have not been fully validated, including RBM15/RBM15B, HAKAI, ZC3H13, ALKBH3, eIF3, hnRNPC, and hnRNPA2B1. Multi-omics information mining based on single-cell sequencing, proteomics, RNA-seq, and m6A methylation sequencing will provide comprehensive information mining for BC tumor sites and cellular models. This unreported regulator in BC is a research gap and therefore has considerable research value, including the relationship between expression abundance and prognosis, diagnostic and therapeutic potential. These can provide a more profound complement to the discovery of new molecules or previous regulatory networks. Thus, continued research is still needed to fully elucidate the role of m6A regulators in mRNA and ncRNA biology.

Finally, aberrant regulation of m6A regulatory proteins is involved in BC drug resistance and tumor immune response. Targeted m6A-based therapies will contribute to the oncological treatment of BC. However, it is worth mentioning that because the regulatory network of m6A modifications is complex and involves multiple signaling molecules and pathways, inhibition of a single molecule may lead to unintended responses. Therefore, for targeted therapies of m6A, or combination strategies with other tumor-targeting drugs, the optimal combination will lead to the best efficacy.

## 5 Conclusion

Overall, m6A is an important mechanism for epigenetic modifications that regulate BC progression. Abnormal m6A modifications trigger the expression, activation, or inhibition of key signaling molecules in critical signaling pathways and the regulatory factors acting on them in BC. M6A-related proteins and their targets show differentially expressed patterns in BC tissue and blood. These m6A-related genes can not only be used as markers for accurate diagnosis, prediction of prognosis, and risk model construction, but also as effective targets for BC treatment.

## Abbreviations

AML, acute myeloid leukemia; ALKBH5, AlkB homolog 5; BC, breast cancer; BCBMs, breast cancer brain metastases ; CSC, cancer stem cell; CSCC, cervical squamous cell carcinoma; ceRNA, competitive endogenous RNA; FTO, fat mass and obesity-associated; GC, gastric cancer; HSCs, hematopoietic stem cells; HBXIP, hepatitis B X-interacting protein; METTL5, methyltransferase-like 5; m6A, N6-methyladenosine; OC, ovarian cancer; OS, overall survival; PD-L1, programmed cell death 1 ligand; PD-1, programmed cell death receptor 1; RBPs, RNA-binding proteins; SNPs, single nucleotide polymorphisms; TNBC, triple-negative breast cancer; TAMs, tumor-associated macrophages.
